# Evaluation of Cellulase, Pectinase, and Hemicellulase Effectiveness in Extraction of Phenolic Compounds from Grape Pomace

**DOI:** 10.3390/ijms252413538

**Published:** 2024-12-18

**Authors:** Natalia Stanek-Wandzel, Alicja Krzyszowska, Magdalena Zarębska, Katarzyna Gębura, Tomasz Wasilewski, Zofia Hordyjewicz-Baran, Magdalena Tomaka

**Affiliations:** 1 Łukasiewicz Research Network-Institute of Heavy Organic Synthesis “Blachownia”, Energetykow 9, 47-225 Kedzierzyn-Kozle, Poland; alicja.krzyszowska@icso.lukasiewicz.gov.pl (A.K.); magdalena.zarebska@icso.lukasiewicz.gov.pl (M.Z.); katarzyna.gebura@icso.lukasiewicz.gov.pl (K.G.); tomasz.wasilewski@urad.edu.pl (T.W.); zofia.hordyjewicz@icso.lukasiewicz.gov.pl (Z.H.-B.); magdalena.tomaka@icso.lukasiewicz.gov.pl (M.T.); 2 Faculty of Applied Chemistry, Casimir Pulaski Radom University, Chrobrego 27, 26-600 Radom, Poland

**Keywords:** enzyme-assisted extraction, cellulase, hemicellulase, pectinase, phenolic compounds, red grape pomace

## Abstract

Grape pomace, the solid residue from winemaking, is a rich source of polyphenolic compounds with significant antioxidant properties. However, the efficient extraction of these valuable compounds remains a challenge. This study focuses on optimizing the conditions for the enzyme-assisted extraction of polyphenolic compounds from red grape pomace using cellulase, hemicellulase, and pectinase. The key variables investigated in this study were enzyme concentration, extraction time, and solid/liquid ratio. The results highlight the importance of selecting enzymes based on target compounds, as different enzymes were found to be more effective for specific phenolic fractions. Hemicellulase was most effective for phenolic acids, cellulase for catechins, and pectinase for anthocyanins. Enzyme-assisted extraction significantly increased the yield of phenolic compounds and resulted in higher total phenolic content and antioxidant activity compared to control samples treated with solid/liquid extraction without enzyme addition. These findings confirm that enzyme-assisted extraction is a promising approach for enhancing the recovery of polyphenolic compounds from grape pomace.

## 1. Introduction

Grape pomace, a by-product of the winemaking process, is a rich source of valuable compounds, particularly phenolic compounds, which when isolated from the pomace, can be reused, for example in the food, pharmaceutical, and cosmetic industries [1]. Phenolic compounds offer a wide range of health benefits, from antioxidant and anti-inflammatory properties to antimicrobial and anticancer effects [2]. However, these compounds can exist in both unbound and bound forms, with the latter being covalently attached to cell wall components and more challenging to extract [3,4].

Traditional extraction methods such as acid or alkaline hydrolysis have been used to release bound phenolic compounds, but these techniques often suffer from drawbacks like long processing times, high solvent consumption, low selectivity, and the potential degradation of thermolabile compounds [3]. Various other techniques, including the use of water, organic solvents, ionic liquids, eutectic solvents, and supercritical fluids, have also been widely employed for the extraction of phenolic compounds [5,6,7]. Each method offers distinct advantages, but they also come with significant limitations. For instance, organic solvents, while effective, often pose environmental and health risks due to their toxicity and the challenges associated with their disposal. One of the main drawbacks of conventional solvent-based extraction is the risk of residual solvents in the final product, which may compromise its safety, particularly in food and pharmaceutical applications [8]. Water, on the other hand, is safe, non-toxic, and sustainable, making it ideal for food-grade uses. Its main drawback is reduced efficiency in extracting certain non-polar phenolics, often necessitating longer extraction times or elevated temperatures [9]. Similarly, methods such as supercritical fluid extraction require specialized equipment and can be energy-intensive [10]. Ionic liquids, despite being a distinctive class of solvents with numerous applications across various fields, face several challenges that hinder their widespread commercial use. Although versatile, their commercial use remains limited due to factors such as high costs, viscosity issues, and potential environmental risks [7]. Eutectic solvents, which are biodegradable and environmentally friendly, provide excellent solvent properties for both polar and non-polar phenolics. However, further research is needed to fully understand their safety, toxicity, and chemical stability [11].

Among the modern techniques, enzyme-assisted extraction (EAE) has shown great promise. EAE involves the use of specific enzymes to hydrolyze the polysaccharides in the plant cell wall, thereby disintegrating the cell structure and releasing the bioactive compounds [12]. One of the key advantages of enzyme-assisted extraction is its efficiency. Enzymes have the ability to selectively target and cleave specific bonds in plant materials, allowing for more targeted and efficient extraction of desired bioactive compounds. This targeted approach can result in higher yields and better purity of the extracted compounds, thus increasing the overall efficiency of the process [12]. Beside efficiency, enzyme-assisted extraction also offers significant environmental benefits over conventional methods. Enzyme-assisted extraction uses aqueous systems, minimizing the use of hazardous chemicals and reducing the environmental impact of the extraction process [13]. In addition, enzyme-assisted extraction exhibits a high degree of selectivity, enabling targeted extraction of specific compounds of interest. This selectivity is particularly beneficial in the food and nutraceutical industries, where it is crucial to maintain the integrity and functionality of bioactive compounds [12]. Moreover, enzyme-assisted extraction can be performed under milder conditions, such as lower temperatures and pH levels, which can help to preserve the structural and functional properties of the extracted biomolecules [13]. Enzymes such as polygalacturonase, xylanase, pectin esterase, cellulase, hemicellulase, amylase, β-galactosidase, protease, 1, 4-glucosidase, tannase, papainase, and tyrosinase are commonly used in EAE. The choice of enzyme depends on the type of plant material and the specific target compounds [14].

The structure of grape pomace cell walls, consisting of an outer pectin-rich layer and an inner cellulose–hemicellulose layer, presents a unique challenge for extracting and recovering valuable polyphenolic compounds [15]. The key to unlocking these compounds lies in the targeted application of specific enzymes, each with a distinct mode of action, to disintegrate the cell wall matrix [16]. By selectively targeting the cell wall polymers, these enzymes facilitate the release of the valuable polyphenolic compounds trapped within, paving the way for their efficient extraction.

In summary, the extraction of phenolic compounds from plant materials is a complex process that requires the careful selection of methods to maximize the yield of the bioactive compounds. This study aims to evaluate the efficiency of enzyme-assisted extraction for the recovery of phenolic compounds from red grape pomace (GP). Specifically, the study focuses on determining the optimal conditions for EAE, including cellulase, hemicellulase, and pectinase concentrations, mass-to-eluent ratios, and extraction times, to maximize the total phenolic content (TPC) and antioxidant activity of the extracts. The study also compares the effectiveness of EAE with the traditional solid–liquid extraction method to highlight the advantages and potential limitations of using enzymes in the extraction process.

## 2. Results and Discussion

### 2.1. Optimization of Enzyme-Assisted Extraction Conditions

**Figure 1:**
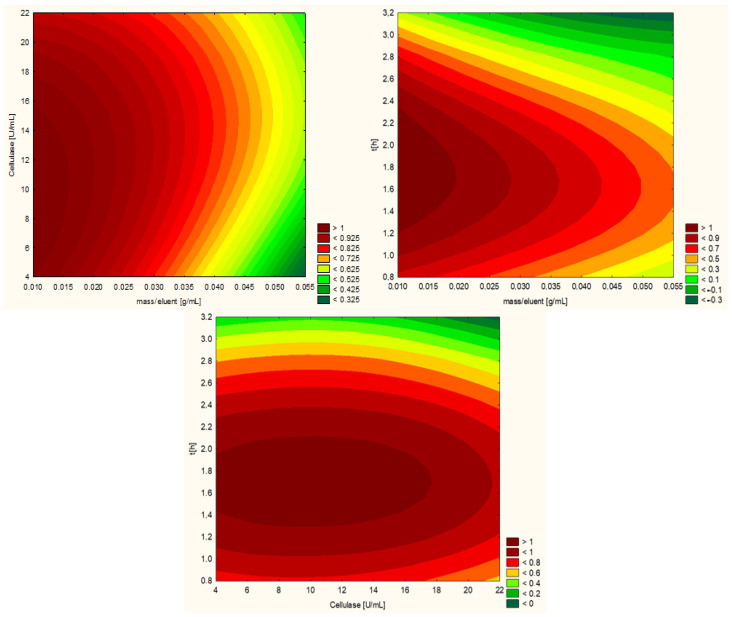
Response surface plots showing the relationships between total phenolic content and antioxidant activity extracts and reaction parameters: extraction time, cellulase amount, and solid-to-liquid ratio value.

The extraction of valuable compounds from grape pomace is influenced by various factors, including pH, temperature, enzyme concentration, extraction time, and the ratio of solids to liquids [17]. Therefore, optimizing the extraction conditions is essential in order to maximize the yield of the polyphenolic fraction. In the present study, the optimization of enzymatic extraction conditions for cellulase, hemicellulase, and pectinase was investigated. According to the manufacturer’s specifications, the optimal pH and temperature conditions (see table in Section 3.3.1) for each enzyme were strictly adhered to as they were predetermined to ensure maximum enzymatic activity. These parameters were considered fixed and were not subject to experimentation in our study. The crucial variables, including enzyme concentration, extraction time, and solid-to-liquid ratio, were investigated to establish the optimal conditions for enhancing the grape pomace extracts’ total phenolic content and antioxidant activity. The Design of Experiments (DoE) approach used a Central Composite Design (CCD) and Response Surface Methodology (RSM). The design consisted of 17 runs, including 3 central points. Each experimental variable (factor) takes on three levels: low (minimum), medium (central value), and high (maximum). The ranges for input variables were as follows: enzyme concentration (5–20 U/mL), extraction time (1–3 h), and solid-to-liquid ratio (1/80–1/20 g/mL). Table 1 presents the planned experiment and the obtained results for cellulase as an example, and Figure 1 graphically displays them on response surface plots. Experimental plan tables for the other enzymes are included in the Supplementary Materials (Tables S2 and S3). The experimental results were then analyzed to establish the optimal conditions for each enzyme, which were subsequently used to prepare the corresponding grape pomace extracts. The optimized conditions for the enzymes were as follows: cellulase—9.5 U/mL, 1.6 h, and 0.25 g/20 mL; hemicellulose—11 U/mL, 2.2 h, and 0.55 g/20 mL; and pectinase—5 U/mL, 1 h, and 0.35 g/20 mL.

The final predictive equation was determined based on the regression coefficients and values of variables, including the ratio of solids to liquids (X_1_), enzyme concentration (X_2_), and extraction time (X_3_) provided in the Table 2 for the cellulase enzyme.

For the cellulase enzyme, the analysis revealed that two linear terms (X_1_ and X_3_) and one quadratic term (X_3_) were significant at a *p*-value less than 0.05. Conversely, all other interaction terms were found to be insignificant (*p* > 0.05). The model predicted a total phenolic content of 1990 mg of GAE/100 g and a DPPH radical scavenging activity of 4054 mg TE/100 g, which closely aligned with the observed values of 1924 mg/100 g GAE (RSD = 2.4) and 4121 mgTE/100 g (RSD = 1.1), respectively. This strong agreement between predicted and experimental results validated the reliability of the model in optimization. In the case of hemicellulase, the quadratic term for the mass-to-eluent ratio and the quadratic term for time were significant in the total phenolic content assay. For the DPPH radical scavenging activity assay, the linear term for the mass-to-eluent ratio, the quadratic term for the mass-to-eluent ratio, and the quadratic term for time were significant. The discrepancies between predicted and observed values were RSD = 3.5 for TPC and RSD = 1.0 for DPPH. For the pectinase enzyme, all tested parameters, including the linear terms for the mass-to-eluent ratio, temperature, and time, as well as the quadratic term for time, were found to be significant in both the TPC and DPPH assays. For pectinase, differences were found for TPC RSD = 5.6 and DPPH RSD = 7.0. The statistically significant results of the test are presented in the Pareto charts, which are included in the Supplementary Materials Figures S3–S5.

### 2.2. Phenolic Profile of Extracts

**Figure 2:**
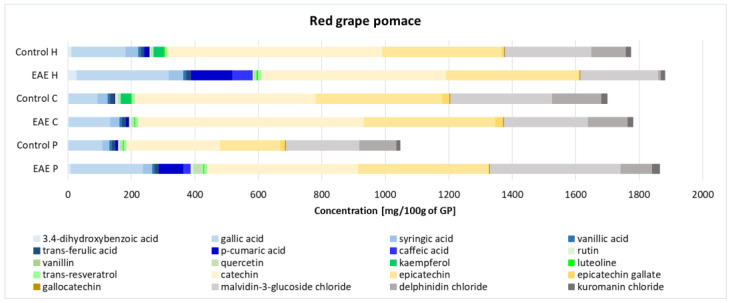
Proportion of phenolic compounds in grape pomace extracts depending on the enzymes used. Blue color—phenolic acids, yellow color—catechins, grey color—anthocyanins, and green color—other flavonoids.

The enzyme-assisted extraction using pectinase (EAE P), cellulase (EAE C), and hemicellulase (EAE H) enzymes yielded varying concentrations of different classes of phenolic compounds (Figure 2). A table with the concentrations of individual compounds is available in the Supplementary Materials (Table S4). Yields were compared with control samples (Control P, Control C, and Control H) that did not undergo enzymatic treatment. The distinct extraction profiles observed with each enzyme can be attributed to their specific mechanisms of action. The use of hemicellulase resulted in the highest extraction of phenolic acids, a class of simple phenolic compounds, such as 3, 4-dihydroxybenzoic acid, gallic acid, syringic acid, vanillic acid, trans-ferulic acid, p-coumaric acid, and caffeic acid. Hemicellulase targets hemicellulose [18], a complex polysaccharide in the plant cell wall, breaking it down and thereby facilitating the release of bound phenolic acids. Cellulase treatment was most effective for extracting flavonoids, particularly catechins, including catechin, epicatechin, epicatechin-3-gallate, and gallocatechin. Cellulase breaks down cellulose, another major component of the plant cell wall [19]. The degradation of cellulose exposes flavonoid-rich cell structures, enhancing the extraction of these more complex phenolic compounds. Pectinase facilitated the highest recovery of anthocyanins, such as malvidin-3-glucoside chloride, delphinidin chloride, and kuromanin chloride. Malvidin-3-O-glucoside content was consistently higher compared to other anthocyanins, suggesting that the enzymes used were particularly efficient in extracting this compound (up to 4 times more than delphinidin chloride and 18 times more than kuromanin chloride). Pectinase degrades pectin, a polysaccharide that holds plant cells together. The breakdown of pectin releases anthocyanins, which are often found in the vacuoles of plant cells and bound to pectic substances [16,20].

The differences in the extraction profiles can be attributed to the specific activities of the enzymes, which target different components of the plant cell wall and facilitate the release of phenolic compounds with varying degrees of complexity. These findings suggest that the choice of enzyme used for the extraction of phenolic compounds from grape pomace can significantly impact the profile and concentration of the extracted compounds.

Previous studies have shown the potential of using enzymes to enhance the extraction of phenolic compounds from various plant materials. For example, the extraction of polyphenols from unripe apples using enzymes, including arabinase, cellulase, β-glucanase, hemicellulase, and xylanase, increased p-coumaric, ferulic, and caffeic acids 8-fold, 4-fold, and 32-fold, respectively [21]. Similarly, the enzymatic release of phenolic compounds from blackcurrant pomace using commercial protease and pectinase increased phenolic amounts, but lower anthocyanin extraction efficiencies were reported [22]. The lower amounts of anthocyanins in the extracts were attributed to the presence of β-glucosidase, β-galactosidase, or α-L-arabinosidase in the multicomponent enzyme preparations, which can release sugar molecules from the anthocyanins, forming unstable aglycones. Studies have also explored the use of specific enzymes, such as α-L-rhamnosidase, to enhance the extraction of target phenolic compounds. The authors of [23] demonstrated that α-L-rhamnosidase could interfere with the pectin–cellulose complex in kinnow peel waste, resulting in the increased extraction efficiency of naringin.

The enzymatic extraction of phenolic compounds from grape by-products has also been studied, with different studies investigating the effectiveness of different enzyme combinations in enhancing the recovery of valuable phytochemicals. One study examined the enzymatic extraction of gallic acid, caffeic acid, quercetin, and trans-resveratrol from Moscato Bianco grape pomace using tanninase, which proved highly effective. In addition, a combination of pectinase and cellulase was shown to increase catechin content in red grape pomace [24]. The extraction of anthocyanins from the Cabernet Sauvignon grape skins using enzymes, including pectinase polygalacturonase and pectin lyase, showed the highest percentage of anthocyanin recovery, exceeding 50% [25]. Interestingly, the use of enzymes did not affect the extraction of anthocyanins from Petit Verdot grapes, probably due to the fact that these grapes had already been enzymatically treated during processing, usually with pectinase [25]. Authors of Ref. [26] observed that the preparation of Lallzyme EX-V, despite lacking cellulase and hemicellulase activities, showed higher efficiency in extracting flavonoids (anthocyanins, flavonol glycosides, and flavan-3-ols) from grape skins than other preparations with these enzymatic activities.

### 2.3. Total Phenolic Content and Antioxidant Activity of Extracts

The study examined the impact of enzyme-assisted extraction on the total phenolic content and antioxidant activity of grape pomace extracts. As presented in Table 3, the findings demonstrated that using enzymes significantly enhanced the extraction of phenolic compounds compared to the control samples. The highest total phenolic content was observed in the cellulase-assisted extract (1924 ± 16 mg GAE/100 g), followed by extracts obtained with hemicellulase and pectinase. Similarly, antioxidant activity, assessed through DPPH and ABTS assays, was higher in the enzyme-treated extracts. The cellulase-assisted extract exhibited the greatest antioxidant capacity in the DPPH assay, while the hemicellulase-assisted extract performed best in the ABTS assay. Enzyme-assisted extraction facilitated the release of phenolic compounds from the plant matrix, enhancing their bioavailability and antioxidant properties.

These findings are consistent with previous studies that reported the benefits of enzymatic extraction in improving the recovery of bioactive phenolic compounds from plant materials. For instance, studies on blackcurrants have shown that the use of enzymes in the extraction process can increase the total polyphenol content by 1.4–2.1 times compared to the control sample. The best results were obtained with the Rohapect^®^ MC enzyme, a pectinolytic enzyme mixture, suggesting its effectiveness in elevating polyphenol content. Moreover, the increased polyphenol content was accompanied by a substantial improvement in the antioxidant activity of the extracts, with the greatest increase of 100% observed with Rohapect^®^ MC. Viscozyme^®^ L, a cellulolytic enzyme mixture, also significantly enhanced antioxidant activity, by 63.4% [27]. Similar findings have been reported for other plant materials. The enzymatic extraction of acerola juice using Celluclast under optimal conditions led to a 35.7% increase in vitamin C, a 9.0% increase in phenolic compounds, and 23.9% and 22.6% increases in antioxidant activity based on DPPH and ABTS, respectively, compared to the control sample [28]. Furthermore, acerola peel extracted with a protease/peptidase enzyme showed the best result for total phenolic content, equivalent to 45.46 mg GA/g DW, along with a significant increase in antioxidant activity, as measured by FRAP, DPPH, and ABTS assays [29]. In a study by [30], various commercial enzyme products, namely Celluclast 1.5 L, Pectinex Ultra, and Novoferm, were evaluated for their effectiveness in releasing phenolic compounds from grape waste. Among the enzymes tested, Novoferm showed the highest antioxidant activity with a value of 90 ± 0.37% after 12 h of treatment. This was followed by Pectinex Ultra and Celluclast 1.5 L, which showed antioxidant activity of 82.9 ± 0.31% and 86.8 ± 0.81%, respectively.

### 2.4. Effect of Enzymes on the Extraction of Reducing Sugars

Enzymatic extraction is a widely employed technique for the recovery of valuable compounds from plant materials. This approach exploits the ability of enzymes to hydrolyze the complex polysaccharides found in cell walls, facilitating the release and diffusion of biomolecules.

The effectiveness of enzymatic extraction can be monitored by measuring the concentrations of reducing sugars liberated during the process. The data presented in Table 4 provide insights into the performance of different enzymes in extracting sugars from grape pomace. The cellulase enzyme appears to be the most effective in releasing glucose, which aligns with its ability to hydrolyze endo-1, 4-β-D-glycosidic bonds in cellulose, lichenin, and barley β-glucans. When applied alone, cellulase breaks down cellulose into smaller oligosaccharides, such as cellotriose and cellohexaose, but does not directly hydrolyze cellobiose or p-nitrophenyl-β-D-glucoside. The glucose detected in the process is likely a result of the further hydrolysis of these oligosaccharides using minor β-glucosidase impurities in the enzyme preparation. Thus, cellulase initiates the breakdown of cellulose, making it a key enzyme for glucose release [16]. Pectinase is particularly effective in liberating fructose and glucuronic acid. Pectinase catalyzes the depolymerisation of pectins by hydrolyzing the α-1 4-galactosidic bonds in polygalacturonates, which are key components of the intercellular matrix in plant cell walls. The degradation of pectin via pectinase leads to a loosening of the cell wall structure, facilitating the release of sugars associated with pectins, including glucuronic acid. The pectic substances were found to be a triad of polysaccharides consisting mainly of galacturonan, rhamnogalacturonan-I, and rhamnogalacturonan-II. In addition, there are other sugars in the side chains, such as glucuronic acid, fucose, glucose, mannose, and xylose [31]. Hemicellulase hydrolyzes hemicelluloses, such as xyloglucans, arabinoxylans, and galactoglucomannans, releasing oligosaccharides and monosaccharides like xylose, arabinose, and mannose. Hemicellulase is generally less effective than pectinase and cellulase, though it shows moderate glucose and glucuronic acid extraction results. This is likely due to its limited specificity for cellulose and its primary focus on hemicellulose structures. However, hemicellulase contributes to the breakdown of the hemicellulose matrix, which indirectly aids in the release of simple sugars from the plant material.

The varying effectiveness of these enzymes can be attributed to the complex and heterogeneous structure of the grape pomace cell wall, which comprises a pectin-rich outer layer and an inner cellulose–hemicellulose matrix. The distinct mechanisms of action of cellulase, pectinase, and hemicellulase, targeting different polysaccharide linkages, explain the differences in the sugar profiles observed.

The data presented in Table 4 underscore the significance of choosing suitable enzymes for the effective extraction of phenolic compounds from grape pomace, as the efficiency of each enzyme depends on the unique composition and structure of the cell wall.

**Figure 3:**
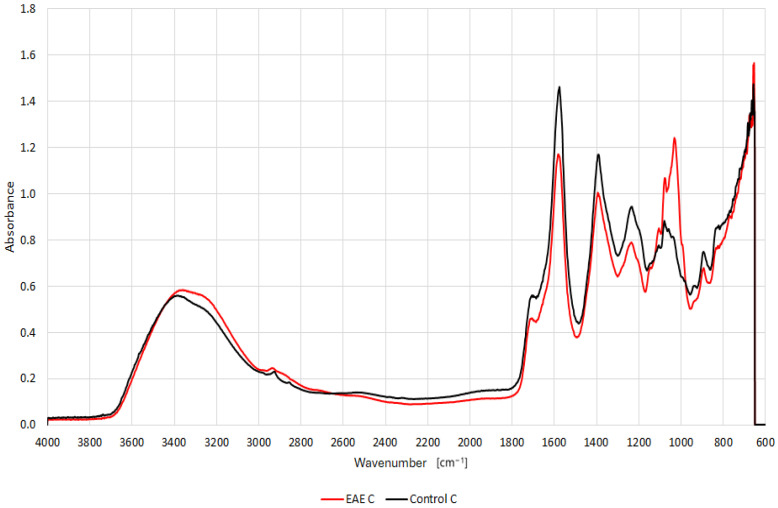
Spectra of extracts after extraction without and with cellulase enzyme for grape pomace.

FTIR analyses confirmed that the enzymes effectively hydrolyzed the complex polysaccharides present in cell walls. Extract samples were compared after extraction with and without the addition of enzymes. An additional strong band at approximately 1026 cm^−1^ was recorded in the FTIR spectra for extracts from pomace grapes. FTIR spectra in the wavenumber between 950 and 1200 cm^−1^ are considered to represent the ‘fingerprint’ region for carbohydrates (in this range, vibrations of C-O and C-C groups are observed) [32]. The same relationship was observed in all tested extracts. Example spectra of extracts after extraction without and with cellulase enzymes for pomace grape are shown in Figure 3. Spectra for the remaining enzymes are provided in the Supplementary Materials (Figures S1 and S2).

## 3. Materials and Methods

### 3.1. Chemicals and Reagents

Methanol of LC-MS grade was supplied by J.T Baker (Phillipsburg, NJ, USA). All standards used were of analytical grade with a purity of ≥99%. Chempur provided sodium citrate and citric acid (Piekary Slaskie, Poland). Ultrapure water with a resistance of less than 18 MΩ cm was produced using the Direct-Q water purification system.

The standards used for identification and quantification were as follows: rutin, syringic acid, vanillic acid, trans-ferulic acid, p-coumaric acid, kaempferol, caffeic acid, 3,4-dihydroxybenzoic acid, vanillin, (+)-catechin (C), (−)-epicatechin (EC), (−)-gallocatechin (GC), (−)-epicatechin 3-gallate (ECG), fructose, D-(+)-xylose, glucose, mannose, glucuronic acid, kuromanin chloride, and delphinidin chloride all obtained from Merck (Darmstadt, Germany); trans-resveratrol from LGC (Teddington, Middlesex, UK); gallic acid, quercetin, ABTS (2,20-azino-bis(3-ethylbenzothiazoline-6-sulfonic acid) diammonium), and Trolox (6-hydroxy-2, 5, 7, 8-tetramethylchromane-2-carboxylic acid) from POL-AURA (Zabrze, Poland); DPPH (2, 2-diphenyl-1-picrylhydrazyl) from Sigma-Aldrich (Saint Louis, MO, USA); and malvidin-3-glucoside chloride from PHYTO LAB (Vestenbergsgreuth, Germany).

Enzymes, namely cellulase from *Aspergillus niger*, hemicellulase from *Aspergillus niger*, and pectinase from *Aspergillus*, were obtained from Sigma-Aldrich (Saint Louis, MO, USA).

### 3.2. Plant Material

Red grape pomace (Léon Millot hybrid varieties: Millardet et Grasser 101 O.P. × Goldriesling × *Vitis rupestris* × *Vitis riparia*) were donated by the Estro Vineyard from Ujazd (Opolskie Voivodeship, Strzelce Opolskie district). GP was mixed with dry ice in a laboratory knife mill (Cutter Mixer R5 Plus, Robot Coupe, Palinges, France), packed in polyethylene plastic bags, frozen, and stored at −20 °C for later use. The dry matter content of the grape pomace samples was 26.3 ± 1.5 and was measured using the thermogravimetric method (Mettler Toledo TGA2 Thermogravimetric Analyzer, Greifensee, Switzerland).

### 3.3. Extraction Procedure

#### 3.3.1. Enzyme-Assisted Extraction

All unbound and bound phenolic compounds from the grape pomace were extracted using enzyme-assisted extraction. The procedure was performed under the optimum extraction conditions, i.e., with an optimum mass-to-eluent ratio, extraction time, and concentration for each enzyme, which were selected using an advanced analytical tool, the Design of Experiments (DoE), conducted in the presented study. For sample preparation, a 10 kg batch of grape pomace was thoroughly mixed to ensure homogeneity. From this, 50 g samples were taken from various locations within the pomace mass, and then crushed and homogenized to create a representative 500 g sample. A portion of the ground grape pomace extract was then mixed with an enzymatic solution containing cellulase (9.5 U/mL), hemicellulase (11 U/mL), and pectinase (5 U/mL), each dispersed in 20 mL of citrate buffer. The mixture was incubated in a BioSan Environmental Shaker-Incubator ES-20/60 and was shaken at 150 rpm for the following times and temperatures: 1.6 h at 37 °C for cellulase, 2.2 h at 40 °C for hemicellulase, and 1 h at 50 °C for pectinase. After incubation, the extract was filtered and analyzed. The enzyme-specific buffer pH and incubation temperatures, as provided by the manufacturer (according to the certificate of analysis from Sigma-Aldrich), were used to ensure efficient enzyme activity (Table 5). All extractions were performed in triplicate.

#### 3.3.2. Solid–Liquid Extraction (SLE)

Unbound phenolic compounds were recovered using the solid–liquid extraction method. The extraction was performed similarly to the enzyme-assisted extraction, with the key difference being that a citrate buffer was used without the addition of enzymes. The extracts were then filtered and analyzed. All extractions were performed in triplicate. This protocol allows control samples to be obtained.

### 3.4. Experimental Design

In this study, the effects of several variables were evaluated using the Design of Experiments statistical methods (Statistica aver. 13.3 software, StatSoft, Tulsa, OK, USA). The variables of each enzyme (cellulase (C), hemicellulase (H), and pectinase (P)) included concentrations of 5, 12.5, and 20 U/mL; mass-to-eluent ratios of 1:20, 1:50, and 1:80 g/mL; and extraction times of 1, 2, and 3 h. The total phenolic compound content and antioxidant activity (DPPH test) of the extracts were measured as the responses of the design experiments. The variables used, with cellulase as an example, are presented in Table 1. For each enzyme, 17 experiments were carried out. The experimental data were analyzed using regression analysis and fitted to an empirical second-order polynomial model, represented by the following equations:

(1) $$y_{k}=\beta_{0}+\beta_{1}x_{1}+\beta_{2}x_{2}+\beta_{3}x_{3}+\beta_{11}x_{1}^{2}+\beta_{22}x_{2}^{2}{+\beta }_{33}x_{3}^{2}+\beta_{12}x_{1}x_{2}+\beta_{13}x_{1}x_{3}+\beta_{23}x_{2}x_{3}$$

where *y_k_* = response variable, *y*_1_ = TPC (mg GAE/100 g), *y*_2_ = DPPH (mg TE/100 g), and *x*_1_, *x*_2_, and *x*_3_ represent the coded independent variables for the mass-to-eluent ratio, concentration, and extraction time of each enzyme, respectively. βo is the model constant.

### 3.5. Analysis of Grape Pomace Extracts

#### 3.5.1. Determination of Phenolic Compounds Using UHPLC-MS/MS

The previously filtered (0.2 µm NY) extract solutions were diluted and separated using an ultra-high performance liquid chromatography (UHPLC) system (Sciex ExionLC AD, AB Sciex, Concord, ON, Canada) with a reverse-phase column (Kinetex 3.5 µm XB-C18 100 Å; 100 × 4.6 mm, Phenomenex, Torrance, CA, USA) according to the procedure detailed in [33]. Detection was carried out with a quadrupole mass spectrometer (4500 QTRAP, AB Sciex, Concord, ON, Canada) equipped with an electrospray ionization source operating in both negative and positive ion modes. Quantification was achieved using a multiple reaction monitoring (MRM) scan mode. All MS/MS transitions are provided in the Supplementary Materials (Table S1), and the spectrometer parameters are as described in publication [34]. Data processing was performed using Analyst software version 1.7.2.

#### 3.5.2. Determination of Sugars Using HPLC-ELSD

The HPLC analyses were carried out using an Ultimate 3000 Dionex HPLC system (Dionex, CA, USA) with ELSD detector (Sedere, France). Sugar standards and sample solutions were separated using a Luna Omega SUGAR column (3 μm, 250 mm × 4.6 mm, 100 Å), maintained at a temperature of 35 °C in a column oven. Analysis was carried out using an isocratic elution, with acetonitrile/water (75:25, *v*/*v*) as the eluents, at a flow rate of 1.0 mL min^−1^.

#### 3.5.3. Fourier Transform Infrared Spectroscopy (FTIR)

The chemical structures of different extracts were characterized by ATR FTIR (Nicolet 6700 FT-IR spectrometer Thermo Scientific (Waltham, MA, USA) with OMNIC™ Series software version 8.0.) using a ZnSe 60° crystal, over a range of 4000–400 cm^−1^. The FTIR spectrum was collected for 32 scans in the reflection mode. Extracts were evaporated to dryness on a vacuum evaporator under reduced pressure (Rotavapor R II, Buchi, Switzerland). Then, 2 mL of water was added to the extract, and all of it was poured onto the crystal and evaporated using a laboratory dryer.

#### 3.5.4. Determination of Total Phenolic Contents

The total phenolic content of grape pomace extracts was determined using the Folin-Ciocalteu assay [35]. To perform the analysis, 80 µL of red grape pomace extract was mixed with 0.2 mL of Folin–Ciocalteu reagent and 0.6 mL of a saturated Na_2_CO_3_ solution. Distilled water was then added to bring the total volume to 4 mL. The mixture was incubated in the dark at room temperature (~23 °C) for 120 min. Absorbance was measured at 765 nm using an HP 8452A spectrophotometer (Hewlett Packard, Palo Alto, CA, USA). TPC was expressed as milligrams of gallic acid equivalents (mg GAE) per 100 g of dry grape pomace weight.

#### 3.5.5. Determination of Antioxidant Properties

Two spectrophotometric methods were employed to evaluate the antioxidant activity of the extracts. The DPPH radical scavenging activity was measured following a modified procedure from [36], while the ABTS radical scavenging activity was assessed using a modified method based on [37]. Absorbance was recorded at 517 nm for DPPH and 734 nm for ABTS using a spectrophotometer. The antioxidant activity results were expressed as Trolox equivalents in milligrams per 100 g of dry grape pomace.

## 4. Conclusions

The results of this study suggest that the choice of enzymatic treatment can have a significant impact on the composition and antioxidant properties of the extracted phenolic compounds. Pectinase, cellulase, and hemicellulase enzymes were found to exhibit different characteristics towards various classes of phenolic compounds, indicating that a combination of these enzymes may be an effective approach to increase extraction efficiency. The use of cellulase, hemicellulase, and pectinase enzymes improved the extraction of phenolic antioxidants, with the cellulase- and hemicellulase-assisted extracts showing the strongest antioxidant activity. These findings highlight the potential of enzymatic extraction techniques to increase the total phenolic content and antioxidant activity of plant-based extracts, such as those derived from grape pomace. The use of enzymatic extraction also allows for the hydrolysis of polysaccharides from the cell wall of the tested plants, which was confirmed by HPLC and FTIR tests. Further research is needed to explore the potential commercial applications of these enhanced phenolic extracts. Future perspectives: To enhance the sustainability and cost-effectiveness of enzymatic extraction processes, a scheme for the reuse of enzymes is proposed. One promising approach is the immobilization of enzymes on solid supports. Immobilized enzymes can be easily separated from the extraction medium and reused multiple times. Techniques such as adsorption, covalent bonding, and entrapment within polymer matrices could also be explored [38]. In particular, immobilized enzymes on porous or functionalized solid matrices can enhance substrate interaction, leading to the improved dissolution or permeabilization of solid structures like plant cell walls. By hydrolyzing polysaccharides or other complex components, immobilized enzymes can release entrapped bioactive compounds, including phenolics. The choice of immobilization method should ensure compatibility with the matrix properties, while factors like enzyme loading, binding efficiency, and support porosity should be optimized for maximum catalytic performance [39]. In processes using free enzymes, ultrafiltration or membrane technologies can be applied to recover enzymes post-extraction [40]. Alternatively, precipitation methods (e.g., using ammonium sulfate) could concentrate and purify enzymes for subsequent reuse. Enzymes should be purified from contaminants, such as residual plant extracts, using techniques like chromatography, dialysis, or other separation methods [41]. Moreover, the performance analysis of both immobilized and recovered enzymes should be conducted to monitor their efficiency and stability across multiple uses. Key parameters to evaluate include activity retention, substrate turnover rates, and any structural modifications resulting from repeated use [38]. Research into novel enzyme immobilization materials, such as nanomaterials or hybrid composites, could further enhance enzyme reusability and catalytic performance [39]. By integrating enzyme immobilization, advanced recovery techniques, and physical enhancement methods, it is possible to significantly reduce production costs while maintaining or even improving the quality and bioactivity of phenolic extracts. Such advancements align with sustainable production goals and pave the way for more eco-friendly and cost-effective enzymatic extraction technologies.

## Figures and Tables

**Table 1:** Variables used in the Design of Experiments for evaluating the effect of cellulase on phenolic extraction from grape pomace.

Run Order	Cellulase U/mL of GP	Mass-to- Eluent Ratio	Extraction Time (h)	TPC (mg GAE/100 g of GP)	DPPH (mg TE/100 g of GP)
1	5	1/80	1	1893 ± 33	3618 ± 67
2	5	1/80	3	1836 ± 37	3320 ± 73
3	20	1/80	1	1784 ± 35	3040 ± 55
4	20	1/80	3	1726 ± 49	2658 ± 57
5	12.5	1/80	2	2128 ± 26	3972 ± 3
6	5	1/50	1	1872 ± 19	3744 ± 41
7	5	1/50	3	1219 ± 25	2353 ± 23
8	20	1/50	1	1889 ± 38	3792 ± 35
9	20	1/50	3	1476 ± 20	2641 ± 77
10	12.5	1/50	2	1971 ± 48	4108 ± 76
11	5	1/20	2	1698 ± 38	3068 ± 8
12	20	1/20	2	1803 ± 2	3080 ± 2
13	12.5	1/20	1	1644 ± 3	3015 ± 25
14	12.5	1/20	3	1233 ± 41	2337 ± 47
15	12.5	1/20	2	1841 ± 25	3173 ± 16
16	12.5	1/20	2	1858 ± 20	3128 ± 7
17	12.5	1/20	2	1845 ± 21	3127 ± 12

**Table 2:** Predictive equation for TPC and DPPH based on regression coefficients and experimental variables.

	β_0_	β_1_X_1_	β_2_X_2_	β_3_X_3_	β_11_(X_1_)^2^	β_22_(X_2_)^2^	β_33_(X_3_)^2^	β_12_X_1_X_2_	β_13_X_1_X_3_	β_23_X_2_X_3_	Sum
Cellulase											
TPC	1457.7	−773.1	24.7	2009.1	206.7	−54.2	−886.5	56.5	−110.7	60.8	1990
DPPH	2049	342.8	437	3129.6	−286.9	−270.8	−1495.0	161.5	−58.5	45.6	4054
Hemicellulase											
TPC	−988	1587.3	792	3584	−535.4	−605	−1584	69.5	−132.9	−330	1927
DPPH	−2641	4668.4	2739	8437	−2428	−1331	−4114	171.2	−253.6	−242	5006
Pectinase											
TPC	2885.2	−289.5	−126	−670.4	98.1	22.5	163.1	−44.9	−75.1	13.5	1976
DPPH	4401.3	55.2	−355.5	−912.7	−26.3	17.5	224.9	−2.2	−136.4	24	3290

**Table 3:** Total phenolic content and antioxidant activity of extracts prepared by enzymatic extraction. The letters next to the numerical values indicate statistically significant differences between groups, determined using ANOVA and Tukey’s post hoc test (*p* < 0.05).

	EAE P	Control P	EAE C	Control C	EAE H	Control H
TPC [mgGAE/100 g dw]	1830 ± 9 ^ab^	1562 ± 5 ^c^	1924 ± 16 ^a^	1717 ± 10 ^b^	1836 ± 5 ^ab^	1608 ± 23 ^c^
DPPH [mgTE/100 g dw]	3650 ± 53 ^c^	3066 ± 1 ^d^	4121 ± 15 ^b^	3360 ±15 ^d^	5075 ± 43 ^a^	3875 ± 44 ^c^
ABTS [mgTE/100 g dw]	7227 ± 105 ^c^	6223 ± 77 ^d^	8138 ± 31 ^a^	7160 ±29 ^c^	7670 ± 39 ^b^	7256 ± 98 ^c^

**Table 4:** Concentrations of sugars and glucuronic acid released from grape pomace by different enzymes. The letters next to the numerical values indicate statistically significant differences between groups, determined using ANOVA and Tukey’s post hoc test (*p* < 0.05).

mg/100 g	EAE P	Control P	EAE C	Control C	EAE H	ControlH
Glucose	7550 ± 129 ^b^	6610 ± 150 ^c^	10,300 ± 312 ^a^	4800 ± 187 ^d^	6680 ± 287 ^c^	6660 ± 238 ^c^
Fructose	7524 ± 279 ^a^	4254 ± 225 ^c^	5022 ± 112 ^b^	5032 ± 251 ^b^	4896 ± 144 ^b^	4860 ± 254 ^b^
Xylose	5240 ± 98 ^b^	4500 ± 85 ^c^	5760 ± 74 ^a^	5530 ± 62 ^ab^	5220 ± 80 ^b^	4730 ± 56 ^c^
Maltose	336 ± 12 ^c^	97 ± 8 ^d^	1420 ± 88 ^a^	45 ± 10 ^d^	488 ± 32 ^b^	76 ± 9 ^d^
Glucuronic acid	7681 ± 55 ^a^	977 ± 12 ^c^	589 ± 26 ^d^	452 ± 27 ^d^	2464 ± 68 ^b^	517 ± 16 ^d^

**Table 5:** Characteristics of the following enzymes: cellulase, hemicellulase, and pectinase.

Enzyme	Optimum pH	Optimum T [°C]	Source
Cellulase	5.0	37 °C	*Aspergillus niger*
Hemicellulase	4.5	40 °C	*Aspergillus niger*
Pectinase	4.0	50 °C	*Aspergillus*

## Data Availability

The original contributions presented in this study are included in the article and Supplementary Materials. Further inquiries can be directed to the corresponding author.

## Supplementary Materials

The following supporting information can be downloaded at: https://www.mdpi.com/article/10.3390/ijms252413538/s1.
