# Supplementary material for: Evaluation of Cellulase, Pectinase, and Hemicellulase Effectiveness in Extraction of Phenolic Compounds from Grape Pomace
Source: Int J Mol Sci. 2024 Dec 18;25(24):13538. doi: 10.3390/ijms252413538 (PMC11677007; doi:10.3390/ijms252413538)
Supplement: Supplementary file 1 [file ijms-25-13538-s001.zip › ijms-3338948-supplementary.pdf]

Table S1. Variables used in the Design of Experiments for evaluating the effect of hemicellulase on phenolic extraction from grape pomace. To ensure effective enzyme activity, an enzyme-specific buffer pH=4.5 and an incubation temperature of 40°C were used, as declared by the manufacturer.

Run Order	Cellulase U / mL of GP	Mass-to-eluent ratio	Extraction time (h)	TPC (mg GAE / 100 g of GP)	DPPH (mg TE / 100 g of GP)
1	5	1/80	1	1387	2722
2	5	1/80	3	1697	3110
3	20	1/80	1	1285	2686
4	20	1/80	3	1622	2770
5	12.5	1/80	2	2492	5126
6	5	1/50	1	1670	3314
7	5	1/50	3	2085	4275
8	20	1/50	1	1655	2760
9	20	1/50	3	1883	3453
10	12.5	1/50	2	2434	5016
11	5	1/20	2	1862	3022
12	20	1/20	2	1939	3027
13	12.5	1/20	1	1648	2739
14	12.5	1/20	3	1733	2806
15	12.5	1/20	2	1701	2944
16	12.5	1/20	2	1716	2973
17	12.5	1/20	2	1688	2958

Results are shown as mean; n = 3.

Table S2. Variables used in the Design of Experiments for evaluating the effect of pectinase on phenolic extraction from grape pomace. To ensure effective enzyme activity, an enzyme-specific buffer pH=4.0 and an incubation temperature of 50°C were used, as declared by the manufacturer.

Run Order	Cellulase U / mL of GP	Mass-to-eluent ratio	Extraction time (h)	TPC (mg GAE / 100 g of GP)	DPPH (mg TE / 100 g of GP)
1	5	1/80	1	2018	3348
2	5	1/80	3	1967	3329
3	20	1/80	1	1968	2497
4	20	1/80	3	1958	2480
5	12.5	1/80	2	1860	2656
6	5	1/50	1	1997	3189
7	5	1/50	3	1778	2725
8	20	1/50	1	1824	2640
9	20	1/50	3	1727	2462
10	12.5	1/50	2	1709	2711
11	5	1/20	2	1593	2486
12	20	1/20	2	1304	1721
13	12.5	1/20	1	1725	2609
14	12.5	1/20	3	1397	1965
15	12.5	1/20	2	1264	1929
16	12.5	1/20	2	1274	1930
17	12.5	1/20	2	1280	1896

Results are shown as mean; n = 3.

Table S3. Concentration of key phenolic compounds in grape pomace extracts, illustrating the impact of different enzymatic treatments (H, C, P) on extraction efficiency.

	EAE P	Control P	EAE C	Control C	EAE H	Control H
3.4-dihydroxybenzoic acid	9.2 ±0.46	1.7 ±0.10	4.4 ±0.18	4.0 ±0.28	27.9 ±1.40	12.3 ±0.74
gallic acid	228.5 ±11.4	108.7 ±6.5	130.4 ±5.2	91.3 ±6.39	290.5 ±14.5	171.1 ±10.2
syringic acid	29.0 ±1.45	21.3 ±1.28	28.3 ±1.13	31.2 ±2.19	45.6 ±2.28	39.9 ±2.40
vanillic acid	8.2 ±0.41	8.1 ±0.49	8.7 ±0.35	8.5 ±0.59	9.8 ±0.49	8.6 ±0.52
trans-ferulic acid	12.0 ±0.60	11.1 ±0.66	11.8 ±0.47	12.7 ±0.89	14.8 ±0.74	10.1 ±0.61
p-cumaric acid	77.6 ±3.88	8.2 ±0.49	10.0 ±0.40	2.8 ±0.19	129.3 ±6.4	16.2 ±0.97
caffeic acid	23.2 ±1.16	nd	nd	nd	65.8 ±3.29	0.8 ±0.05
rutin	7.9 ±0.40	7.2 ±0.43	8.1 ±0.32	9.2 ±0.64	3.5 ±0.18	5.0 ±0.30
vanillin	0.8 ±0.04	1.0 ±0.06	1.1 ±0.04	1.5 ±0.10	1.4 ±0.07	1.2 ±0.07
quercetin	30.8 ±1.54	7.2 ±0.43	7.7 ±0.31	6.7 ±0.47	7.2 ±0.36	6.1 ±0.37
kaempferol	nd	nd	nd	32.1 ±2.26	nd	32.1 ±1.92
luteoline	3.9 ±0.20	2.7 ± 0.16	2.8 ±0.11	2.8 ±0.20	3.7 ±0.19	3.0 ±0.18
trans-resveratrol	9.3 ±0.46	7.9 ±0.48	9.1 ±0.36	9.2 ±0.65	11.3 ±0.57	8.2 ±0.49
catechin	475.3 ±23.7	295.4 ±17.7	711.0 ±28.4	570.3 ±39.9	581.7 ±29.1	676.8 ±40.6
epicatechin	410.6 ±20.5	189.7 ±11.3	414.4 ±16.5	399.2 ±27.9	418.3 ±20.9	376.4 ±22.5
epicatechin 3-gallate	2.0 ±0.10	14.7 ±0.88	24.3 ±0.97	21.3 ±1.49	1.8 ±0.09	6.9 ±0.41
gallocatechin	3.1 ±0.15	2.9 ±0.18	3.2 ±0.13	2.9 ±0.21	2.9 ±0.14	2.7 ±0.16
malvidin-3-glucoside chloride	410.6 ±20.5	230.8 ±13.8	263.5 ±10.5	320.5 ±22.4	245.2 ±12.2	273.0 ±16.4
delphinidin chloride	98.9 ±4.9	117.5 ±7.0	125.5 ±5.0	155.9 ±10.9	8.3 ±0.41	109.1 ±6.55
kuromanin chloride	23.2 ±1.16	10.2 ±0.61	15.2 ±0.61	16.9 ±1.18	11.1 ±0.56	13.7 ±0.82

Table S4. The MS/MS transitions and compounds' characteristic parameters for quantified compounds.

Compound	Precursor ion m/z	Main product ions MS^2 m/z	Ionization mode	DP, V	EP, V	CE, V	CXP, V
Gallic acid-1	168.9	124.8	ESI -	-40	-10	-20	-7
Gallic acid-2	168.9	78.9	ESI -	-40	-10	-28	-7
Rutin-1	608.9	299.9	ESI -	-150	-10	-50	-9
Rutin-2	608.9	270.9	ESI -	-150	-10	-82	-9
Quercetin-1	300.9	151.0	ESI -	-90	-10	-30	-7
Quercetin-2	300.9	179.0	ESI -	-90	-10	-26	-7
Syringic acid-1	196.9	120.9	ESI -	-60	-10	-24	-7
Syringic acid-2	196.9	181.9	ESI -	-60	-10	-18	-7
Vanillic acid-1	166.9	107.9	ESI -	-5	-10	-26	-9
Vanillic acid-2	166.9	123.0	ESI -	-5	-10	-16	-7
trans-ferulic acid-1	192.9	133.9	ESI -	-60	-10	-24	-7

trans-ferulic acid-2	192.9	178.0	ESI -	-60	-10	-18	-7
p-cumaric acid-1	162.8	118.9	ESI -	-45	-10	-22	-7
p-cumaric acid-2	162.8	93.0	ESI -	-45	-10	-44	-7
Caffeic acid-1	178.9	135.0	ESI -	-65	-10	-24	-7
Caffeic acid-2	178.9	134.0	ESI -	-65	-10	-34	-7
3.4-dihydroxybenzoic acid-1	152.8	109.0	ESI -	-55	-10	-20	-5
3.4-dihydroxybenzoic acid-2	152.8	107.9	ESI -	-55	-10	-34	-5
trans-Resveratrol-1	226.9	185.0	ESI -	-75	-10	-26	-5
trans-Resveratrol-2	226.9	143.0	ESI -	-75	-10	-36	-7
kaempferol-1	284.9	184.9	ESI -	-100	-10	-38	-7
kaempferol-2	284.9	117.0	ESI -	-100	-10	-56	-7
vanillin-1	150.8	135.9	ESI -	-40	-10	-18	-7
vanillin-2	150.8	91.9	ESI -	-40	-10	-28	-7
Luteoline-1	284.9	132.9	ESI -	-95	-10	-46	-7
Luteoline-2	284.9	132.0	ESI -	-95	-10	-68	-7
(+)-Catechin /(-)-Epicatechin-1	290.9	139.0	ESI +	81	10	23	10
(+)-Catechin /(-)-Epicatechin-2	290.9	123.0	ESI +	81	10	47	10
(-)-Gallocatechin-1	306.9	138.9	ESI +	71	10	21	10
(-)-Gallocatechin-2	306.9	288.8	ESI +	71	10	11	12
Malvidin-3-glucoside chloride-1	493.0	331.0	ESI +	96	10	31	14
Malvidin-3-glucoside chloride-2	493.0	315.0	ESI +	96	10	67	14
(–)-Epicatechin 3-gallate-1	306.9	288.8	ESI +	71	10	21	10
(–)-Epicatechin 3-gallate-2	306.9	163.0	ESI +	71	10	11	12
Delphinidin chloride-1	302.9	228.9	ESI +	121	10	45	10
Delphinidin chloride-2	302.9	69.0	ESI +	121	10	91	6
Curomanin chloride-1	449.0	287.0	ESI +	91	10	33	12
Curomanin chloride-2	449.0	128.0	ESI +	91	10	107	12

Collision energy (CE), declustering potential (DP), entrance potential (EP), and collision cell exit potential (CXP).

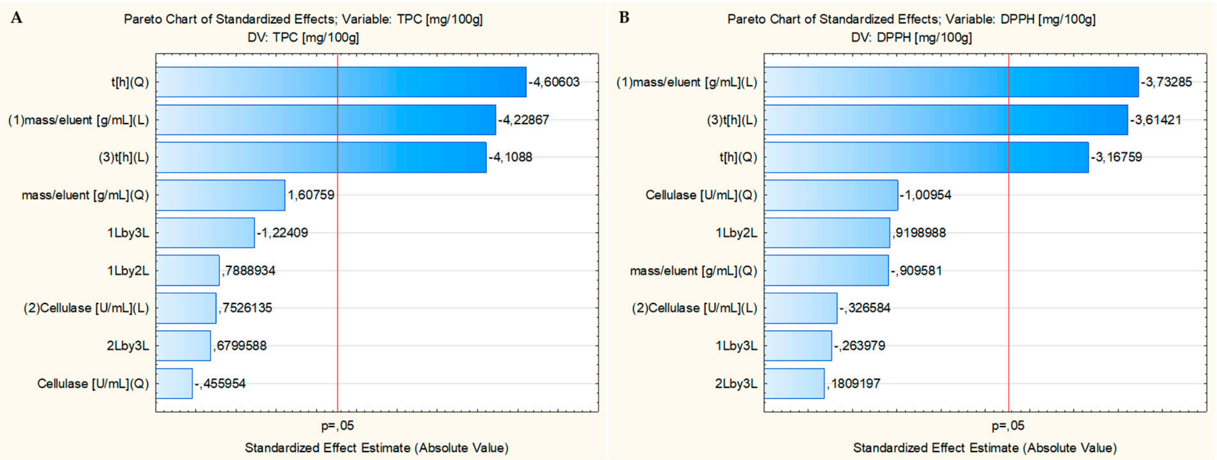

Figure S1. Pareto Chart of standardized effects for the variable TPC [mg/100g], and DPPH [mg/100g] for the enzyme cellulase. The vertical line indicates statistical significance with more than 95% confidence.

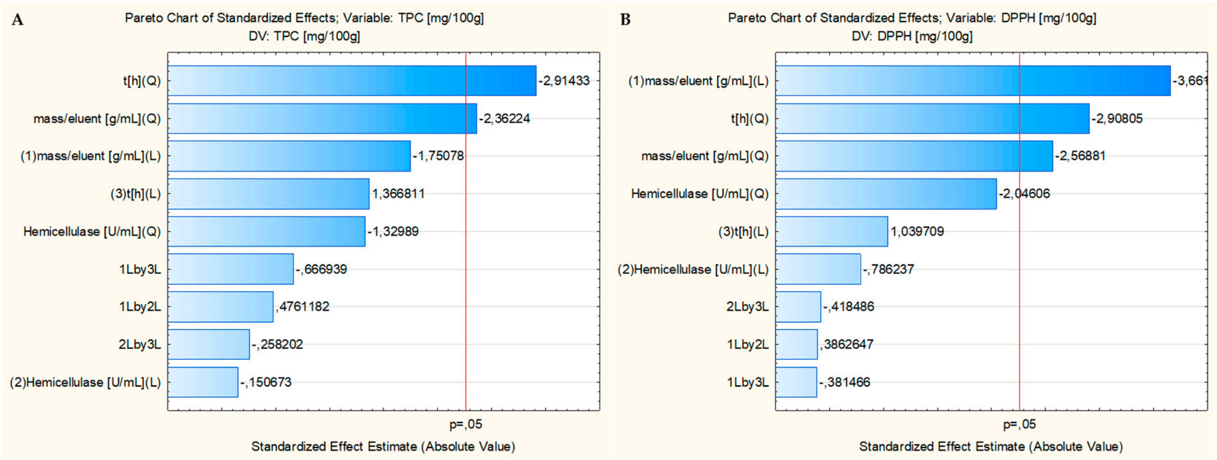

Figure S2. Pareto Chart of standardized effects for the variable TPC [mg/100g], and DPPH [mg/100g] for the enzyme hemicellulase. The vertical line indicates statistical significance with more than 95% confidence.

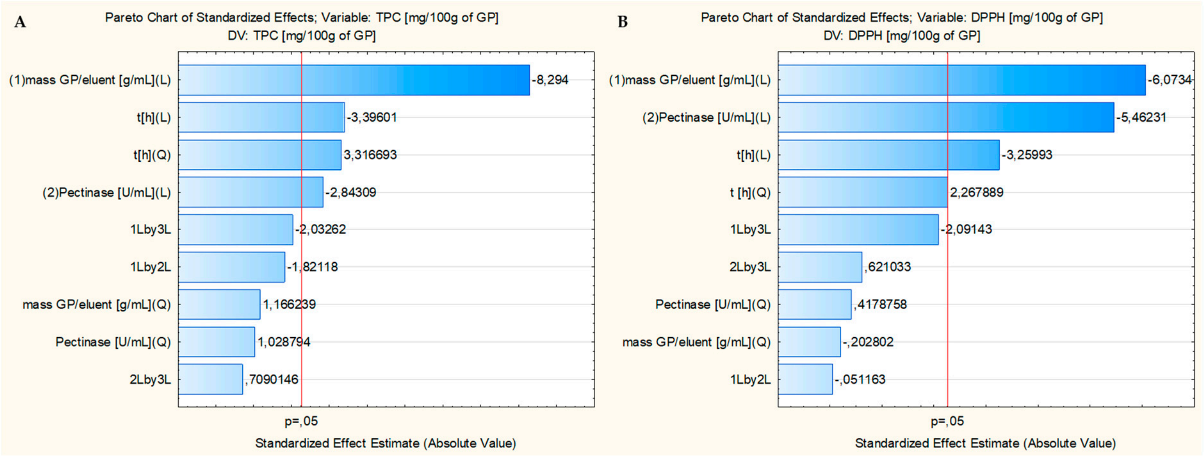

Figure S3. Pareto Chart of standardized effects for the variable TPC [mg/100g], and DPPH [mg/100g] for the enzyme pectinase. The vertical line indicates statistical significance with more than 95% confidence.

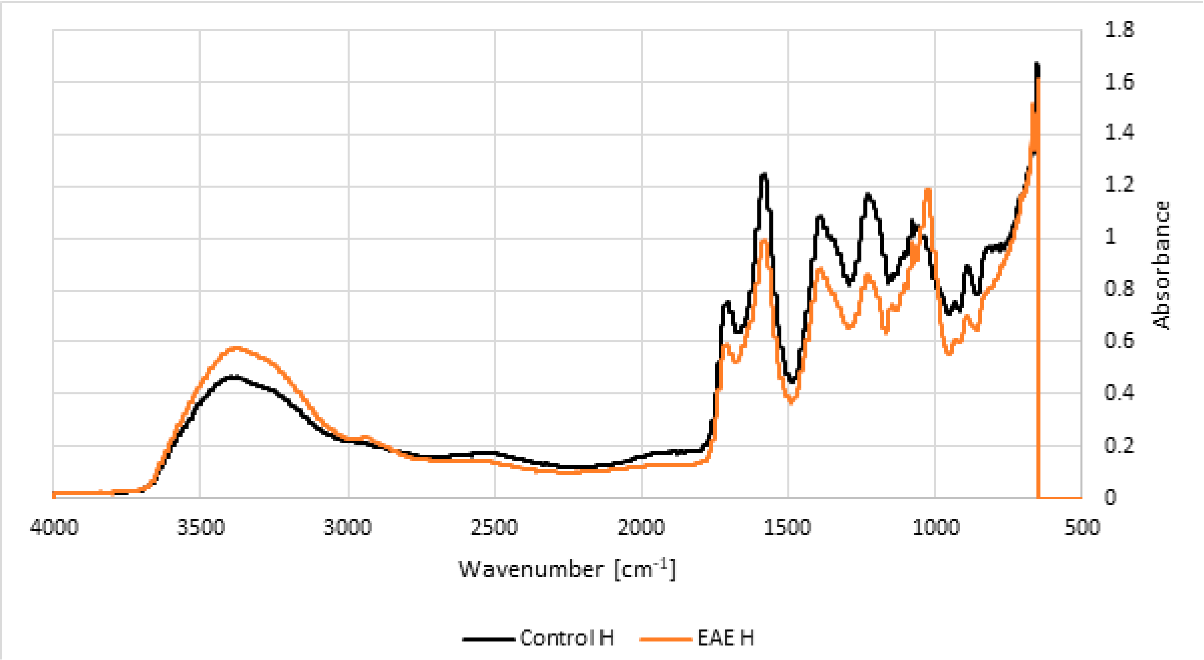

Figure S4. Spectra of extracts after extraction without and with hemicellulase enzyme for grape pomace.

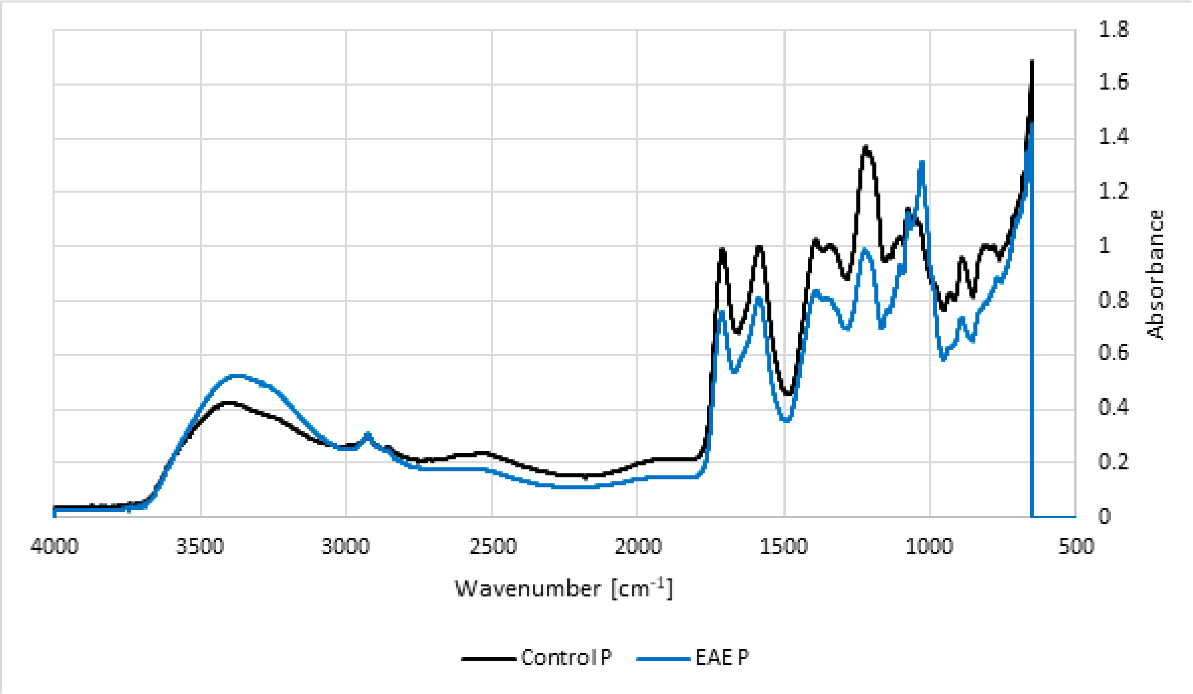

Figure S5. Spectra of extracts after extraction without and with pectinase enzyme for grape pomace.
